# Comparative study on the efficacy of menadione, zoledronic acid, and calcitriol in combination with PKP for the treatment of osteoporotic vertebral compression fractures in extremely elderly patients

**DOI:** 10.1097/MD.0000000000044952

**Published:** 2025-10-03

**Authors:** Huajun Yu, Hongtao Hou, Yan Jin

**Affiliations:** a Orthopedics Department, Zhejiang Academy of Traditional Chinese Medicine, Hangzhou, Zhejiang, China; b Radiology Department, Zhejiang Academy of Traditional Chinese Medicine, Hangzhou, Zhejiang, China; c Medical Department, Zhejiang Academy of Traditional Chinese Medicine, Hangzhou, Zhejiang, China.

**Keywords:** combination therapy, extremely elderly, menadione, osteoporotic vertebral compression fracture, PKP, zoledronic acid

## Abstract

This study compares the efficacy and safety of menadione and zoledronic acid, both used in conjunction with calcitriol, in patients with osteoporotic vertebral compression fractures following percutaneous kyphoplasty (PKP) in an extremely elderly population. Patients with osteoporotic vertebral compression fractures (OVCF) were assigned into 4 groups. The basic treatment group received PKP and calcitriol for osteoporosis treatment. Experimental group 1 received menadione capsules in addition to the control treatment; experimental group 2 received zoledronic acid; experimental group 3 received both menadione soft capsules and zoledronic acid. The outcomes were assessed at 3, 6, and 12 months postoperatively, measuring visual analog scale (VAS) scores, Oswestry disability index (ODI), bone mineral density, anterior vertebral height ratio, correction of Cobb angle, inflammatory markers, biochemical markers of bone turnover, risk of refracture, and adverse drug reactions. At 3, 6, and 12 months postoperatively, both VAS and ODI scores in experimental groups 1, 2, and 3 were significantly lower than baseline and the basic treatment group. However, group 3 showed significantly lower scores than both groups 1 and 2 (all *P* < .05). Bone mineral density in experimental groups 1, 2, and 3 increased significantly compared to baseline and was higher than the basic treatment group, with no significant difference between groups 1 and 2, but group 3 showed significantly higher values than both groups 1 and 2 (all *P* < .05). Levels of SVCAM-1, SICAM-1, UNTX, and SBAP in experimental groups 1, 2, and 3 decreased significantly at 3, 6, and 12 months post-treatment compared to baseline and the basic treatment group, with group 3 showing significantly lower levels than groups 1 and 2 (all *P* < .05). The refracture rates within 12 months were 10.00% (4/40), 7.50% (3/40), 7.50% (3/40), and 2.50% (1/40) for groups 1, 2, 3, and the basic treatment group, respectively, with no significant difference (*P* > .05). In extremely elderly patients with osteoporotic vertebral compression fractures, the combination of calcitriol with menadione soft capsules and zoledronic acid post-PKP significantly improves bone mineral density, promotes new bone formation, and effectively alleviates pain, with good short-term safety.

## 1. Introduction

Osteoporosis (OP) is a systemic skeletal disease characterized by disrupted bone metabolism and imbalanced bone remodeling due to various causes. Its pathological features include a decrease in bone mineral density (BMD) and deterioration of bone microstructure, significantly increasing the risk of fractures among affected individuals. The most severe consequence of OP is osteoporotic fractures, with osteoporotic thoracolumbar compression fractures (OTCF) being the most prevalent, accounting for nearly 50% of all OP fractures. OTCF primarily affects the elderly, particularly postmenopausal women, and its incidence increases with age. Epidemiological surveys indicate that as China experiences population aging, the incidence of OTCF has reached 58.1%, with approximately 23.8% of patients losing some degree of functional ability and about 3.1% succumbing within a year.^[1,2]^

Extremely elderly patients often present with multiple chronic underlying conditions. In cases of osteoporosis-related vertebral compression fractures (OVCF), opting for non-surgical treatment may lead to complications such as deep vein thrombosis, pressure ulcers, and pulmonary infections due to prolonged bed rest, ultimately resulting in serious and irreversible consequences, potentially endangering life. Currently, the most common minimally invasive surgical procedure for treating OVCF is percutaneous kyphoplasty (PKP), which involves injecting bone cement into the compressed vertebra to restore its height. This technique utilizes the reinforcing effect of the cement to rapidly reshape the fractured vertebra and maintain normal vertebral height.

Implementing an anti-OP treatment plan post-PKP can effectively sustain surgical outcomes, reduce the risk of vertebral height loss, mitigate pain recurrence, and lower the probability of adjacent vertebral fractures. However, monotherapy has shown suboptimal results, prompting the pursuit of rational and effective combination therapies in clinical practice. Menadione, a newly developed anti-OP medication, primarily containing vitamin K2, exerts a bidirectional beneficial regulatory effect on OP by promoting bone formation while inhibiting bone resorption.^[3]^ Calcitriol, an active form of vitamin D, regulates serum calcium and phosphorus levels.^[4]^ Zoledronic acid, a third-generation bisphosphonate, effectively inhibits bone resorption, reduces the risk of re-fractures, and improves functional scores in osteoporotic vertebral fractures.^[5]^

Despite the distinct pharmacological mechanisms of these 3 drugs, research on their combined effects in extremely elderly OCVF patients post-surgery is limited. This study aims to investigate the therapeutic efficacy and significance of combining menadione, calcitriol, and zoledronic acid with PKP in the treatment of extremely elderly patients with OCVF.

## 2. Research objects and methods

### 2.1. Patients

This study was designed as a single-center clinical trial. Patients with OVCF treated at the Department of Spine Surgery, between May 2022 and May 2024, were included. The trial was approved by the Ethics Committee of Zhejiang Academy of Traditional Chinese Medicine, and all participants provided written informed consent.

Inclusion criteria:

1. Patients with primary OVCF in the thoracolumbar or lumbar spine, occurring in a single segment within the past 2 weeks.
2. MRI findings showing low signal intensity on T1-weighted images and high signal intensity on T2-weighted images.
3. Patients aged over 80 with a bone mineral density T-score ≤ −2.5, or a history of fragility fractures, presenting with lower back pain, localized tenderness over spinous processes, and/or vertebral percussion pain.

Exclusion criteria:

1. Secondary vertebral fractures or OP due to malignant tumors, infections, or other diseases.
2. Presence of neurological dysfunction prior to or following surgery.
3. Rupture of the posterior wall of the vertebral body.
4. Presence of intervertebral space stenosis.
5. Allergy to menadione, calcitriol, or zoledronic acid.
6. New fractures following PKP, a history of trauma, incomplete clinical information, or other reasons precluding study inclusion.

### 2.2. Surgical procedures

Patients were placed in a prone position, and the target vertebra was identified using X-ray and MRI. Preoperative localization was performed using Kirschner wires and C-arm fluoroscopy, with markings made on the skin. After standard disinfection and draping, local anesthesia was administered, followed by a percutaneous puncture. Under C-arm guidance, a guide needle was inserted at the outer upper margin of the vertebral arch root for accurate positioning. A cannula was then gradually advanced, stopping 2 mm before the posterior vertebral cortex while monitoring the patient’s response. The guide needle was removed, leaving the working cannula in place. A bone tunnel was created using a screw propeller under C-arm guidance, followed by the insertion of an inflatable balloon into the vertebral body. Contrast was injected to inflate the balloon, restoring the vertebral body to its normal shape. After balloon removal, a cavity for bone cement was created, and bone cement was mixed and injected at low pressure (2–5 mL) to fill the space. Finally, all instruments were removed, and the incision was sutured.

### 2.3. Postoperative treatment and grouping

Patients were retrospectively assigned to 4 groups according to the postoperative anti-OP treatment they had received: experimental group 1 (calcitriol + menadione), experimental group 2 (calcitriol + zoledronic acid), experimental group 3 (calcitriol + menadione + zoledronic acid), and a basic treatment group (calcitriol alone).

In the basic treatment group, second-generation cephalosporins were routinely administered 24 hours post-surgery to prevent infection. Patients were mobilized and began weight-bearing activities 24 hours after the procedure without analgesics. Postoperative anti-OP therapy included daily oral calcium carbonate D3 tablets (1.5 g calcium carbonate, equivalent to 600 mg of calcium, and vitamin D3 125 IU; Batch Numbers: 202103021, 202206020; Approval Number: GuoYaoZhunZi H20183358, produced by Tongda Pharmaceutical Co., Ltd., Shanxi Province) and calcitriol soft capsules (0.25 mcg per capsule; Batch Numbers: 03210806, 03220604; Approval Number: GuoYaoZhunZi H20213982, produced by Sichuan Guowei Pharmaceutical Co., Ltd.), administered at 0.25 mcg twice daily.

In Experimental Group 1, patients received menadione soft capsules (15 mg per capsule; Batch Numbers: 220881A, 220979A; Registration Number: GuoYaoZhunNing J 2015113, produced by Nippon Co., Ltd.), 15 mg orally 3 times per day, in addition to the basic treatment group treatment.

Experimental Group 2 received zoledronic acid (Approval Number: GuoYaoZhunZi H20113138; Specification: 5 mg per vial), administered as an intravenous infusion of 5 mg diluted in 500 mL of 0.9% sodium chloride solution, once annually.

Experimental Group 3 combined the treatments of menadione soft capsules and zoledronic acid, following the same dosages and regimens as Groups 1 and 2.

All 4 groups continued anti-OP therapy for 12 months post-surgery. The use of glucocorticoids, bisphosphonates, and Zhuanggu tablets was prohibited during the treatment period. Follow-ups were conducted via telephone or outpatient visits at 3, 6, and 12 months postoperatively.

### 2.4. Observation indicators

#### 2.4.1. Baseline information

Demographic and clinical data, including gender, age, underlying comorbidities, and body mass index (BMI), were collected for all groups.

#### 2.4.2. Visual analog scale (VAS)

Patients assessed their pain severity using a visual analog scale (VAS) ranging from 0 to 10, where 0 indicated no pain and 10 represented the most severe pain. VAS scores were recorded at baseline (before treatment) and at 3, 6, and 12 months post-treatment.

#### 2.4.3. Oswestry disability index (ODI)

Improvements in daily living activities were assessed using the ODI, which evaluated pain severity, individual physical functions (lifting, sitting, standing, walking), and overall functional capacity (self-care, sexual activity, social interactions, and travel). A score of 0 indicated normal function, while higher percentages (closer to 100%) reflected greater functional impairment. ODI scores were recorded at baseline and at 3, 6, and 12 months post-surgery.

#### 2.4.4. Bone mineral density detection

Bone mineral density (BMD) and bone mineral content at the hip were measured for each subject using dual-energy X-ray absorptiometry (ASY-00409, Hologic, Marlborough) by the same physician. A T-score was calculated, with measurements taken at baseline and at 3, 6, and 12 months post-treatment.

#### 2.4.5. Comparison of imaging indicators

Radiographic evaluations were conducted before treatment and 3 months post-treatment to assess changes in anterior vertebral height ratio and Cobb angle correction.

#### 2.4.6. Determination of inflammatory response and biochemical indicators of bone turnover

Three milliliters of venous blood were collected from the patient’s elbow in the morning at 6 and 12 months prior to treatment. After centrifugation at 3000 rpm for 10 minutes (centrifugal radius of 15 cm), serum was separated and stored at −80°C. Inflammatory markers, including serum soluble vascular cell adhesion molecule-1 (SVCAM-1), soluble intercellular adhesion molecule-1 (sICAM-1), and biochemical markers of bone turnover such as urinary N-telopeptides of type I collagen (UNTX) and serum bone-specific alkaline phosphatase (sBAP), were measured using enzyme-linked immunosorbent assay (ELISA) kits (purchased from Shanghai Enzyme-linked Biotechnology Co. Ltd, Shanghai, China), following the manufacturer’s protocols.

#### 2.4.7. Re-fracture risk and adverse drug reactions

The recurrence rate of fractures within 12 months and adverse reactions such as nausea, vomiting, fever, skin itching, and arthralgia were recorded for all groups. Criteria for re-fracture included imaging diagnoses indicating adjacent vertebral body fractures 12 months after PKP.

### 2.5. Statistical methods

Data were analyzed using SPSS 22.0. Continuous variables following a normal distribution were presented as mean ± standard deviation (x̄ ± s). An independent sample *t*-test was used for inter-group comparisons, while categorical data were expressed as counts (%). The χ^2^ test was applied for group comparisons, with *P* < .05 considered statistically significant.

## 3. Results

### 3.1. Comparison of baseline data among 4 patient groups

A total of 160 patients were enrolled in the study, with 40 patients in each of the 3 experimental groups and the basic treatment group. No significant differences were observed in baseline characteristics such as gender, age, body mass index, comorbidities, and injury site across the 4 groups (*P* > .05). In addition, the baseline data of vertebral compression degree and Cobb angle were also comparable among the 4 groups (*P* > .05). These results indicate that the baseline conditions of all groups were well balanced, ensuring the reliability of subsequent comparisons (see Table 1).

**Table 1:** Comparison of general data of patients.

Project	Control group	Test group 1	Test group 2	Test group 3	*t/*χ^2^	*P*
Age (yr)	83.31 ± 2.29	84.55 ± 1.63	81.17 ± 1.29	82.09 ± 0.45	0.33	.88
Gender (male/female)	19/21	18/22	21/19	20/20	0.64	.91
Injury site [cases (%)]						
Lumbar vertebra	21 (52.5)	20 (50.0)	18 (45.0)	18 (45.0)	0.57	.75
Sternal vertebra	19 (47.5)	20 (50.0)	22 (55.0)	22 (55.0)		
BMI (kg/m^2^)	21.55 ± 0.45	19.71 ± 0.77	22.02 ± 0.21	22.49 ± 0.51	0.51	.89
Comorbidities [cases (%)]						
Diabetes	10 (25.0)	9 (22.5)	11 (27.5)	12 (30.3)	0.33	.72
Hypertension	7 (17.5)	11 (27.5)	9 (22.5)	10 (25.0)		
COPD	7 (17.5)	7 (17.5)	8 (20.0)	11 (27.5)		

### 3.2. Changes in VAS for pain within one year post-operation

No significant differences in VAS scores were observed among the 4 groups prior to surgery (*P* > .05). Postoperatively, VAS scores in experimental groups 1, 2, and 3 were lower than pretreatment levels and significantly reduced compared to the basic treatment group at 3, 6, and 12 months. There was no significant difference between experimental groups 1 and 2; however, group 3 exhibited significantly lower scores compared to both groups 1 and 2 (all *P* < .05). Detailed statistically significant differences are presented in Table 2.

**Table 2:** VAS comparison of patients (x ± s, score).

VAS	Control group (n = 40)	Test group 1 (n = 40)	Test group 2 (n = 40)	Test group 3 (n = 40)	*t/*χ^2^
Pretreatment	7.19 ± 1.33	6.93 ± 2.51	7.22 ± 1.12	7.31 ± 1.54	0.715
3 mo after surgery	3.87 ± 1.91*	3.01 ± 1.57*,†	2.98 ± 1.35*,†	2.51 ± 1.77*,†,‡	3.433
6 mo postoperatively	3.12 ± 1.15*	2.53 ± 1.85*,†	2.69 ± 1.44*,†	2.03 ± 1.29*,†,‡	7.271
12 mo postoperatively	2.78 ± 1.67*	1.72 ± 1.45*,^b^	1.89 ± 1.53*,†	0.97 ± 1.81*,†,‡	8.353

* Compared with that before treatment, *P* < .05.

† Compared with the control group, *P* < .05.

‡ Compared with experimental groups 1 and 2, *P* < .05.

### 3.3. Changes in ODI within one year post-surgery

There were no statistically significant differences in ODI scores among the 4 groups prior to surgery (all *P* > .05). Postoperatively, ODI scores in experimental groups 1, 2, and 3 were lower than their pretreatment levels and significantly reduced compared to the basic treatment group at 3, 6, and 12 months. No significant differences were observed between experimental groups 1 and 2; however, group 3 exhibited significantly lower scores compared to both groups 1 and 2 (all *P* < .05). Detailed statistically significant differences are presented in Table 3.

**Table 3:** Comparison of ODI in patients (x ± s, score).

ODI	Control group (n = 40)	Test group 1 (n = 40)	Test group 2 (n = 40)	Test group 3 (n = 40)	*t*/χ^2^
Pretreatment	73.35 ± 7.17	74.52 ± 6.35	71.69 ± 6.88	75.21 ± 5.35	0.871
3 mo after surgery	37.87 ± 4.91*	33.01 ± 2.57*,†	32.08 ± 5.13*,†	28.87 ± 3.45*,†,‡	7.273
6 mo postoperatively	26.77 ± 2.85*	21.51 ± 3.14*,†	21.08 ± 2.15*,†	17.66 ± 2.87*,†,‡	9.118
12 mo postoperatively	18.32 ± 3.87*	14.54 ± 2.62*,†	15.09 ± 2.43*,†	10.25 ± 1.96*,†,‡	14.015

ODI = Oswestry disability index.

* Compared with that before treatment, *P* < .05.

† Compared with the control group, *P* < .05.

‡ Compared with experimental groups 1 and 2, *P* < .05.

### 3.4. Changes in bone mineral density in patients within one year post-operation

No significant differences in lumbar bone mineral density were observed among the 4 groups prior to surgery (*P* > .05). Postoperatively, lumbar bone mineral density in experimental groups 1, 2, and 3 was higher than pretreatment levels and significantly greater compared to the basic treatment group at 3, 6, and 12 months. There were no significant differences between experimental groups 1 and 2; however, group 3 exhibited significantly higher values compared to both groups 1 and 2 (all *P* < .05). Detailed statistically significant differences are presented in Table 4.

**Table 4:** Comparison of bone mineral density of patients (x ± s, g/cm^2^).

Bone density	Control group (n = 40)	Test group 1 (n = 40)	Test group 2 (n = 40)	Test group 3 (n = 40)	*t*/χ^2^
Pretreatment	0.66 ± 0.14	0.71 ± 0.25	0.69 ± 0.22	0.72 ± 0.51	0.527
3 mo after surgery	0.70 ± 0.51*	0.74 ± 0.29*,†	0.75 ± 0.16*,†	0.80 ± 0.14*,†,‡	2.303
6 mo postoperatively	0.75 ± 0.17*	0.79 ± 0.21*,†	0.80 ± 0.25*,†	0.86 ± 0.33*,†,‡	4.167
12 mo postoperatively	0.82 ± 0.15*	0.89 ± 0.25*,†	0.89 ± 0.17*,†	0.94 ± 0.13*,†,‡	7.012

* Compared with that before treatment, *P* < .05.

† Compared with the control group, *P* < .05.

‡ Compared with experimental groups 1 and 2, *P* < .05.

### 3.5. Comparison of imaging indices in patients within one year post-operation

No significant differences in anterior vertebral height loss or Cobb angle were observed among the 4 groups prior to surgery (*P* > .05). Similarly, no significant differences were found in vertebral height loss or Cobb angle correction between experimental groups 1, 2, and 3 at 3, 6, and 12 months postoperatively (all *P* > .05), as shown in Table 5.

**Table 5:** Comparison of imaging indexes of patients (x ± s, g/cm^2^).

Index	Time	Control group (n = 40)	Test group 1 (n = 40)	Test group 2 (n = 40)	Test group 3 (n = 40)	*t*/χ^2^
Vertebral height loss (mm)	Pretreatment	0.34 ± 0.04	0.35 ± 0.15	0.33 ± 0.19	0.32 ± 0.22	0.439
	3 mo after surgery	0.12 ± 0.11*	0.13 ± 0.21*,†	0.12 ± 0.09*,†	0.14 ± 0.14*,†,‡	0.313
	6 mo postoperatively	0.13 ± 0.07*	0.15 ± 0.22*,†	0.14 ± 0.15*,†	0.14 ± 0.03*,†,‡	0.268
	12 m postoperatively	0.14 ± 0.05*	0.16 ± 0.02*,†	0.16 ± 0.15*,†	0.14 ± 0.04*,†,‡	0.415
Cobb angle of kyphosis	Pretreatment	25.16 ± 3.14	24.71 ± 4.21	25.65 ± 2.93	26.61 ± 4.25	0.377
	3 mo after surgery	8.71 ± 2.54*	7.92 ± 3.39*,†	8.14 ± 3.06*,†	7.88 ± 2.15*,†,‡	0.452
	6 mo postoperatively	8.64 ± 3.37*	8.02 ± 2.75*,†	7.96 ± 2.65*,†	7.86 ± 3.13*,†,‡	0.519
	12 mo postoperatively	8.84 ± 3.25*	7.89 ± 3.43*,†	7.89 ± 2.67*,†	7.94 ± 3.45*,†,‡	0.734

* Compared with that before treatment, *P* > .05.

† Compared with the control group, *P* > .05.

‡ Compared with test groups 1 and 2, *P* > .05.

### 3.6. Comparison of biochemical indicators of inflammatory response and bone turnover in patients

Before treatment, there were no significant differences in inflammatory markers (sVCAM-1, sICAM-1) or biochemical markers of bone turnover (uNTX, sBAP) among the 4 groups (*P* > .05). At 3, 6, and 12 months post-treatment, levels of sVCAM-1, sICAM-1, uNTX, and sBAP in experimental groups 1, 2, and 3 were lower than pretreatment levels and significantly lower than those in the basic treatment group. No significant differences were observed between experimental groups 1 and 2, while group 3 exhibited significantly lower levels compared to both groups 1 and 2 (all *P* < .05). These statistical differences are presented in Table 6.

**Table 6:** Comparison of biochemical indicators of inflammation and bone turnover between the 2 groups of patients (x̄±s).

Index	Time	Control group (n = 40)	Test group 1 (n = 40)	Test group 2 (n = 40)	Test group 3 (n = 40)	*t*/χ^2^
sVCAM-1 (ng/mL)	Pretreatment	921.21 ± 33.25	933.35 ± 31.17	925.34 ± 32.06	911.78 ± 34.15	0.519
	3 mo after surgery	822.34 ± 21.14*	801.21 ± 22.11*,†	793.54 ± 26.09*,†	761.88 ± 21.14*,†,‡	3.543
	6 mo postoperatively	752.34 ± 25.12*	721.23 ± 22.17*,†	729.41 ± 20.35*,†	704.12 ± 22.17*,†,‡	4.573
	12 mo postoperatively	652.15 ± 27.05*	611.04 ± 29.23*,†	615.86 ± 22.25*,†	577.35 ± 26.17*,†,‡	4.365
sICAM-1 (ng/mL)	Pretreatment	301.16 ± 23.24	312.75 ± 24.51	309.43 ± 22.43	343.64 ± 24.17	0.539
	3 mo after surgery	291.45 ± 28.12*	279.92 ± 25.44*,†	273.31 ± 23.16*,†	257.69 ± 28.43*,†,‡	5.756
	6 mo postoperatively	285.57 ± 23.17*	251.43 ± 22.65*,†	249.26 ± 22.35*,†	217.36 ± 23.13*,†,‡	9.272
	12 mo postoperatively	236.75 ± 23.45*	217.58 ± 23.13*,†	221.65 ± 22.43*,†	201.27 ± 23.47*,†,‡	7.583
sBAP (ng/mL)	Pretreatment	15.21 ± 0.33	14.11 ± 0.52	14.36 ± 0.29	15.75 ± 0.37	0.485
	3 mo after surgery	12.47 ± 0.44*	10.54 ± 0.49*,†	10.34 ± 0.56*,†	9.18 ± 0.15*,†,‡	11.693
	6 mo postoperatively	10.51 ± 0.31*	8.53 ± 0.25*,†	8.87 ± 0.63*,†	7.26 ± 0.23*,†,‡	7.282
	12 mo postoperatively	8.75 ± 0.25*	7.25 ± 0.33*,†	7.49 ± 0.62*,†	6.84 ± 0.35*,†,‡	14.810
uNTX (nmol BCE/mmol Cr)	Pretreatment	45.26 ± 13.24	44.31 ± 14.45	51.39 ± 14.83	49.21 ± 14.33	0.863
	3 mo after surgery	8.71 ± 2.54*	7.92 ± 3.39*,†	8.14 ± 3.06*,†	7.88 ± 2.15*,†,‡	8.172
	6 mo postoperatively	23.14 ± 8.27*	20.12 ± 6.95*,†	20.44 ± 7.15*,†	18.27 ± 6.63*,†,‡	9.911
	12 mo postoperatively	22.34 ± 7.24*	19.91 ± 7.29*,†	18.17 ± 6.54*,†	16.21 ± 7.77*,†,‡	11.591

* Compared with that before treatment, *P* > .05.

† Compared with the control group, *P* > .05.

‡ compared with test groups 1 and 2, *P* > .05.

### 3.7. Comparison of incidence of re-fracture and adverse drug reactions in patients

The incidence of recurrent fractures within 12 months was 10.00% (4/40) in treatment group 1, 7.50% (3/40) in both groups 2 and 3, and 2.50% (1/40) in the basic treatment group, with no statistically significant difference (*P* > .05). The incidence of adverse drug reactions, including nausea, vomiting, skin pruritus, and arthralgia, was 10.00% (4/40) in groups 1 and 3, and 7.50% (3/40) in group 2, while the basic treatment group had an adverse reaction rate of 12.50% (5/40). There were no significant differences in the total incidence of adverse drug reactions among the 4 groups (*P* > .05).

## 4. Discussion

### 4.1. Enhanced efficacy of combined treatment with calcitriol, menadione, and zoledronic acid after PKP in extremely elderly patients

Extremely elderly individuals often present with severe OP, making them highly susceptible to fragility fractures. Once these patients experience vertebral compression fractures, they face significant complications, which are major contributors to disability and mortality.^[2]^ Traditional open surgery can achieve favorable outcomes, but it involves substantial trauma, and recovery in older adults tends to be slow. Consequently, minimally invasive surgical approaches are increasingly favored.^[6]^

With advancements in minimally invasive techniques, PKP has emerged as a novel method for vertebral reinforcement, gaining traction in the clinical treatment of osteoporotic thoracolumbar compression fractures (OTCF). PKP involves the injection of bone cement into the compressed vertebral body, maintaining mechanical stability and reducing height loss. This procedure can partially restore vertebral height, effectively correct kyphotic deformities caused by height loss, and alleviate pressure on surrounding tissues and nerves, which is vital for long-term spinal height maintenance and rapid pain relief.^[7]^ Additionally, PKP is performed under local anesthesia, offering high safety with minimal postoperative complications, such as nausea and vomiting, and shorter hospital stays.

However, some studies have indicated that PKP may lead to bone loss and exacerbate OP. Furthermore, the dispersion of bone cement can alter the stress balance in the spine and paravertebral muscles, particularly in the thoracolumbar region, which bears much of the spinal load during movement. This situation can increase the risk of new fractures in the injured vertebra and adjacent bodies post-surgery.^[8,9]^ Therefore, enhancing bone density and reducing the incidence of postoperative re-fractures is a pressing clinical concern.

Our findings suggest that in extremely elderly OTCF patients, the use of calcitriol in conjunction with menadione or zoledronic acid, or a combination of all 3, significantly increases bone mineral density compared to pretreatment levels and monotherapy with calcitriol (*P* < .05). Notably, the combination of all 3 agents resulted in the most pronounced increase in bone density (*P* < .05). At all postoperative time points, the combination group demonstrated lower VAS and ODI scores compared to the control and two-drug combination groups. This indicates that the combination of calcitriol, menadione, and zoledronic acid may more effectively reduce bone resorption, alleviate postoperative discomfort, and decrease the occurrence of vertebral re-fractures.

Although radiographic parameters such as vertebral anterior height ratio and Cobb angle improved after PKP in all groups, no significant intergroup differences were observed (Table 5). This apparent inconsistency with the superior clinical efficacy of combination therapy may be explained by several factors. First, the amount of bone cement injected during PKP was controlled within a relatively narrow range, thereby minimizing variability in imaging outcomes. Second, individual anatomical differences, including baseline vertebral morphology, collapse degree, and bone quality, might have influenced the degree of radiographic correction independently of drug therapy. Third, the pharmacological agents mainly act on bone metabolism and remodeling, which manifest more prominently in pain relief, functional recovery, and biochemical markers rather than in short-term imaging changes. Thus, the lack of significant imaging differences does not contradict the observed clinical benefits of combination therapy.

### 4.2. Combined use of calcitriol, menadione, and zoledronic acid alleviates inflammatory response After PKP in extremely elderly patients

Serum uNTX, a marker of total type I collagen N-terminal cross-links, is produced by the degradation of bone collagen by osteoclasts and is minimally affected by dietary intake, making it an effective indicator of osteoclast activity and bone resorption.^[10]^ Serum bone-specific alkaline phosphatase (sBAP), one of the 6 isoenzymes of alkaline phosphatase, serves as a strong marker of osteoblast activity and bone formation, with its levels positively correlated with the rate of bone formation.^[11]^ Monitoring the dynamic changes in uNTX and sBAP can provide insights into overall bone loss and turnover, aiding in fracture risk prediction.

Our study found that the combination of calcitriol with menadione and zoledronic acid effectively promotes bone formation and inhibits bone resorption, thereby reversing the natural course of systemic bone loss caused by surgery and preventing re-fractures of the injured vertebra and adjacent bodies. Additionally, soluble intercellular adhesion molecule-1 (sICAM-1) and soluble vascular cell adhesion molecule-1 (sVCAM-1) are adhesion factors primarily expressed in the extracellular matrix and intercellular spaces, mediating adhesion between cells and the extracellular matrix, which can trigger inflammatory responses that affect local blood supply to fractures, leading to further inflammatory damage at the fracture site.^[12]^ Our findings indicate that the combination therapy significantly alleviates the body’s inflammatory response, creating a favorable internal environment for tissue repair and accelerating fracture healing.

Patients in the combined medication group exhibited significantly reduced pain scores alongside marked increases in bone density, with imaging and inflammatory and bone metabolic indicators confirming these effects. This efficacy may be attributed to the dual regulatory role of menadione in modulating both bone formation and resorption, effectively achieving a balance. It participates in the transcriptional regulation mediated by osteocalcin and foreign material receptors during bone formation, enhancing osteocalcin production and calcium salt deposition, thus increasing bone calcium content.^[3]^ Zoledronic acid stimulates bone differentiation and significantly inhibits bone resorption while also modulating central nervous system function and enhancing muscle strength, which improves balance and coordination and reduces fall risk. Calcitriol promotes the differentiation and maturation of osteoprogenitor cells and stimulates osteoblast activity, collectively exerting synergistic effects that may improve long-term outcomes and reduce the incidence of re-fractures after PKP.

### 4.3. Refracture rate and potential confounding factors

Although no statistically significant differences were found in refracture rates among the 4 groups, the lowest incidence was observed in the basic treatment group (2.5%). This unexpected trend may have been influenced by several confounding factors. First, differences in exposure dose and medication adherence could have affected treatment efficacy in the extremely elderly population. Second, follow-up deviations might exist, as minor fractures could have been underreported or treated outside our hospital. Third, bone cement leakage during PKP, which may alter local stress distribution and increase the risk of adjacent vertebral fractures, cannot be entirely excluded. These factors may partly explain the paradoxical findings and highlight the need for larger multicenter studies with longer follow-up and standardized monitoring of bone cement leakage and drug compliance to further validate our results.

## 5. Limitations

This study has several limitations. First, it was a retrospective, single-center study with a relatively small sample size, which may restrict the generalizability of the results. Second, all observation indicators were limited to 12 months after surgery; therefore, the long-term efficacy and safety of combining calcitriol, menadione, and zoledronic acid after PKP in extremely elderly patients remain uncertain. We acknowledge this as an important limitation and plan to conduct extended follow-up in future work, including monitoring refracture rates beyond 2 years and continuous assessment of BMD and functional outcomes. Third, patient grouping was based on the actual treatment received rather than randomization, which may have introduced selection bias. Furthermore, medication adherence, exposure dose differences, and procedural factors such as bone cement leakage were not fully assessed, all of which may influence refracture outcomes. Future multicenter, large-sample studies with longer follow-up and stricter evaluation of drug compliance and cement leakage are warranted to validate and expand these findings.

## 6. Conclusion

In conclusion, our findings suggest that the combination of calcitriol, menadione, and zoledronic acid after PKP significantly improves bone mineral density, reduces pain, and enhances functional recovery in extremely elderly patients with osteoporotic vertebral compression fractures. The triple therapy also demonstrates beneficial effects in modulating bone turnover markers and alleviating inflammatory responses, with an acceptable short-term safety profile. Although no statistically significant differences were observed in refracture rates, confounding factors such as exposure dose, follow-up deviations, and bone cement leakage may have influenced the outcomes. Taken together, the results support the potential clinical value of combined pharmacological therapy following PKP, while highlighting the need for further high-quality, long-term studies to confirm its effectiveness and safety in this fragile patient population.

## Author contributions

**Conceptualization:** Huajun Yu, Hongtao Hou, Yan Jin.

**Data curation:** Huajun Yu, Hongtao Hou, Yan Jin.

**Formal analysis:** Huajun Yu, Hongtao Hou, Yan Jin.

**Investigation:** Huajun Yu, Hongtao Hou, Yan Jin.

**Methodology:** Hongtao Hou, Yan Jin.

**Software:** Yan Jin.

**Supervision:** Huajun Yu, Hongtao Hou, Yan Jin.

**Validation:** Huajun Yu, Hongtao Hou.

**Visualization:** Huajun Yu, Yan Jin.

**Writing – original draft:** Huajun Yu, Yan Jin.

**Writing – review & editing:** Huajun Yu, Yan Jin.
